# Arbuscular Mycorrhizal Fungi-Mediated Modulation of Physiological, Biochemical, and Secondary Metabolite Responses in Hemp (*Cannabis sativa* L.) under Salt and Drought Stress

**DOI:** 10.3390/jof10040283

**Published:** 2024-04-12

**Authors:** Haipeng Yuan, Hao Si, Yunshu Ye, Qiuyan Ji, Haoyu Wang, Yuhong Zhang

**Affiliations:** 1 Key Laboratory of Forestry Plant Ecology of Ministry of Education, Northeast Forestry University, Harbin 150040, China; linwan363320733@163.com (H.Y.); sh1359237270@163.com (H.S.); 18445171189@163.com (Y.Y.); jy19997503300@163.com (Q.J.); www763400876@163.com (H.W.); 2 Heilongjiang Provincial Key Laboratory of Ecological Utilization of Forestry-Based Active Substances, Harbin 150040, China

**Keywords:** arbuscular mycorrhizal fungi, salt stress, drought stress, *Cannabis sativa* L., interaction effects

## Abstract

The increasing impact of global climate change has resulted in adversity stresses, like salt and drought, gradually becoming the main factors that limit crop growth. Hemp, which contains numerous medicinal active components and multiple bioactive functions, is widely used in the agricultural, industrial, and medical fields, hence promoting the rapid development of related industries. Arbuscular mycorrhizal fungi (AMF) can establish a symbiotic relationship with 80% of vascular plants. This symbiosis promotes host plant growth, regulates plant physiology and biochemistry, facilitates secondary metabolite synthesis, and enhances resistance to abiotic stresses. However, the effects of salt stress, drought stress, and AMF interaction in hemp are not well understood. In this study, to investigate this, we performed a study where we cultured hemp that was either inoculated or uninoculated with *Funneliformis mosseae* and determined changes in effective colonization rate, growth, soluble substances, photosynthesis, fluorescence, ions, and secondary metabolites by cultivating hemp under different concentrations of NaCl (0 mM, 100 mM, and 200 mM) and different soil moisture content (45%, 25%, and 15%). The results showed that salt, drought stress, or salt–drought interaction stress all inhibited colonization rate after stress, plant growth, mainly due to ion toxicity and oxidative damage. Inoculation with *F. mosseae* effectively alleviated plant growth inhibition under 100 mM NaCl salt stress, drought stress, and salt–drought interaction stress conditions. It also improved osmoregulation, photosynthetic properties, fluorescence properties, and ion homeostasis, and promoted the accumulation of secondary metabolites. However, under 200 mM NaCl salt stress conditions, inoculation with *F. mosseae* negatively affected plant physiology, biochemistry, and secondary metabolite synthesis, although it did alleviate growth inhibition. The results demonstrate that there are different effects of salt–drought interaction stress versus single stress (salt or drought stress) on plant growth physiology. In addition, we provide new insights about the positive effects of AMF on host plants under such stress conditions and the effects of AMF on plants under high salt stress.

## 1. Introduction

With increasing global climate change, adversity stresses, such as salt and drought, are becoming major constraints on crop growth and development [1,2]. Hemp (*Cannabis sativa* L.), as an important cash crop, has a wide range of applications in the fields of textiles, medicines, and health foods, etc. [3,4]. However, in the presence of deteriorating soil conditions and increasing meteorological disasters, the growth and yield of hemp are under serious threat. Salt stress causes the accumulation of salts in the soil, disrupting osmotic regulation inside and outside cells. This disruption affects the normal physiological and metabolic activities of plants, resulting in a negative impact on crop yield and quality [5]. At the same time, drought stress leads to soil moisture shortage, which increases physiological stress and further aggravates the survival pressure on plants [6]. In practice, salt stress and drought stress often coexist, creating salt–drought interaction stress [7]. This compound stress condition initiates more severe challenges for plant growth and development [8].

In search of adaptive strategies that can effectively mitigate plant adversity stress, in recent years, more and more studies have focused on the potential role of arbuscular mycorrhizal fungi (AMF) in increasing plant stress tolerance [9,10,11,12]. AMF establishes a symbiotic relationship with plants and plays a crucial role in this process by forming a web-like mycorrhizal structure known as arbuscular mycorrhizae (AM) in the host plant’s root system. AM plays a multifaceted and positive role in plants, which not only enhances antioxidant enzyme activities [13], reduces lipid membrane peroxidation [11], increases the content of soluble substances [14], and promotes photosynthetic rate and other gas exchange-related properties [15], but also contributes to ionic element uptake [9], regulates the root hormone transport and biosynthetic pathways in plant roots [16], increases the expression of resistance genes [9], and improves the structure of bacterial communities in soil [17], among others. Notable AMF is considered the most reliable component of the soil microbiota for increasing the content of plant secondary metabolites due to its broad host range [18]. Research has demonstrated that AM significantly increases the content of terpenoids, phenols, and nitrogenous compounds in plants [19,20,21]. This emphasizes the critical role of AMF in increasing host plant resistance and maintaining ecosystem function. However, there is still a relative lack of systematic and in-depth research on the response mechanism of AMF to salt, drought, and salt–drought interaction stresses, and on the effects of AMF on plant physiology, biochemistry, as well as the synthesis of secondary metabolites in the important crop of hemp.

Plant secondary metabolites play a crucial role in the regulation of physiological and biochemical processes in plants under adverse environments, in addition to the role of AMF [22]. These metabolites not only facilitate the growth and development process of plants but also regulate their stress tolerance [23]. However, there is a lack of detailed research on the effects of AMF on the synthesis and accumulation of these secondary metabolites and their physiological function under stress conditions, such as salt, drought, and salt–drought interaction.

This study aimed to conduct and in-depth investigation into the mechanism of AMF’s role in salt, drought, and salt–drought interaction stresses in hemp, with special attention to its regulatory effects on plant secondary metabolites. A comprehensive study was conducted to systematically explore the effects of salt, drought, and salt–drought interaction stress on the growth, development, physiology, biochemistry, and secondary metabolism of hemp. This investigation aimed to uncover the interactions between AMF and hemp and to understand how these interactions influence the synthesis and accumulation of secondary metabolites in the plant. This study will provide new perspectives on the physiological adaptive mechanisms of plants under multiple adversity conditions and effective support for the development of scientific and rational agricultural production measures in the future. Moreover, this research on hemp is expected to provide useful insights for the improvement of stress tolerance in other cash crops.

## 2. Materials and Methods

### 2.1. Growth Conditions

Hemp seeds were harvested from Bama town, Guangxi (107°15′ E, 24°8′ N) and used in this study. The AMF strain used in this experiment was *Funneliformis mosseae* (https://www.uniprot.org/taxonomy/27381, accessed on 10 May 2022), obtained from the Institute of Plant Nutrition and Resources, Academy of Agricultural and Forestry Sciences, Beijing, China. The AMF inoculants included a mixture comprising soil substrate, spores, clover (*Trifolium repens* L.) mycorrhizal fragments, and *F. mosseae* mycelia. The number of spores was 700–800 spores10 g^−1^.The pot experiments were conducted in a greenhouse with a relative humidity of 60 ± 5%, an ambient temperature of 25 ± 1 °C, and a light cycle of 16 h of light (800 mmol m^−2^ s^−1^) followed by 8 h of darkness. After the pre-test, various treatment groups were established, as outlined in Table 1. Apart from single salt and drought stresses, salt–drought interaction stresses were also applied. Each stress treatment had groups with inoculated AMF (AM) and those without inoculation (NM).

Each treatment consisted of three replicates, with a pot with two hemp plants as one replicate. Filled seeds were selected, sterilized with 75% alcohol, washed three times with distilled water, and placed in autoclaved (121 °C, 2 h) sand to raise seedlings. After 7 days, the seedlings were transferred into plastic pots (16 cm diameter and 17 cm height) containing autoclaved soil mixture (soil–sand 3:1, *v*:*v*). Each pot received either 20 g of inoculants for the inoculation treatment or 20 g of sterilized inoculants for the non-inoculation treatment. To maintain a consistent microbial biota, 30 mL of mycorrhizal propagule-free inoculant, filtered through a 0.25 μm filter membrane, was added to the non-inoculated treatment. The inoculant was added 2 cm below the seed. At 55 days after transplanting, treatment started, and the treatment continued for 23 days. Treatment groups included the following: (1) Control group: Maintained 45% soil moisture content. (2) Salt stress treatment group: Received 300 mL of salt solution every 3 days, applied 5 times in total. At the same time, to avoid salt stress on hemp in the S2 treatment group, 300 mL of 100 mM NaCl was added for the first time, followed by 300 mL of 200 mM NaCl each time. (3) Drought stress treatment group: At the beginning of the treatment, the soil was fully watered until saturation, and then water was controlled until the soil water content reached the objective value. (4) Salt–drought interaction stress treatment group: The last application of salt solution was followed by water control until the soil water content reached the objective value. The hemp was harvested after the treatment was completed.

### 2.2. Determination of Colonization Rate, Mycorrhizal Dependence, Biomass, and Relative Water Content

The colonization rate of mycorrhizal before stress (colonization rate) and after stress (CRAS) was measured using 30 fresh root segments of 1 cm length. Root segment fragments were stained with Tepan Blue (0.05%) according to Phillips et al. [24], and the colonization rate was calculated using the following formula described by Lin et al. [25]: the AMF colonization rate (%) = D (colonized root length/observed root length) × 100%. Each of the above treatments was replicated three times. The stained hemp root segments were observed using a microscope (DM4000B, Leica, Wetzlar, Germany). Mycorrhizal dependence (MD) was calculated using the following equation described by Takács et al. [26]:

MD (%) = (dry weight of AM plants − dry weight of NM plants)/dry weight of AM plants × 100%

At the end of the treatment, the height (Height) and stem diameter (SD) of the plants were measured using a straightedge and vernier caliper. The number of effective blades (NB) of the plants was measured using the counting method. Plants were collected from each treatment group, washed in distilled water, and blotted dry with absorbent paper, and then fresh weight (FW) was measured. The plants were dried at a temperature of 80 °C until constant weight and dry weight (DW) was measured. Leaf relative water content (RWC) determination was performed according to the method of Fan et al. [27]. Each of the above treatments was replicated three times.

### 2.3. Determination of Malondialdehyde, Proline, Soluble Sugar, and Soluble Protein Content

Malondialdehyde (MDA) content was determined by the thiobarbituric acid method, i.e., thiobarbituric acid (0.6% 1 mL) was used to extract malondialdehyde (MDA) from fresh leaves (200 mg), and the absorbance was measured at the wavelengths of 600, 532, and 450 nm [28,29,30]. Measurement of proline (Pro) content was by the acid indan method [31]. Determination of soluble sugars (SS) was by the anthrone colorimetric method [32]. Determination of soluble protein (SP) content was by the Coomassie Brilliant Blue method [33]. The absorbance of the sample solution was measured using a spectrophotometer (UV-2600, Shimadzu, Kyoto, Japan). Each of the above treatments had three replicates.

### 2.4. Determination of SPAD and Photosynthetic Parameters

The SPAD values of the plant leaves were determined using a chlorophyll analyzer (TYS-A, Shandong Jinke Lida Instrument Co., Ltd., Jining, China) with three replications for each treatment. Meanwhile, plant leaf photosynthetic parameters were determined at 1000 μmol m^−2^ s^−1^ using a portable photosynthesis measurement system (LI-6400XT, LI COR, Lincoln, NE, USA). Measurements were taken on 29 April 2023, from 9:00 a.m. to 11:00 a.m. In each treatment, the third fully expanded leaf at the top of the plant was measured for relevant indicators. Each treatment was replicated three times. Measurements included net photosynthetic rate (Pn), stomatal conductance (Gs), intercellular carbon dioxide concentration (Ci), and transpiration rate (Tr), as well as the calculation of leaf water use efficiency (WUE = Pn/Tr).

### 2.5. Determination of Fluorescence Parameters

A portable modulated chlorophyll fluorometer (PAM-2500, Walz, Erlangen, Germany) was used to dark-adapt the apical third fully expanded leaf of plants under different treatments by dark-adapted clamps for 0.5 h. Then, the maximum quantum yield of PSII (Fv/Fm), the actual photochemical efficiency (φPS(II)), the coefficient of photochemical quenching (qP), the coefficient of nonphotochemical quenching (NPQ), and relative electron transfer number rate (ETR) were determined. Each treatment was replicated three times.

### 2.6. Measurement of Ion Content

Elemental analysis of Na^+^, K^+^, Ca^2+^, and Mg^2+^ in plant leaves was performed using an inductively coupled plasma optical emission spectrometry analyzer (ICP-OES) (avio200, Perkinelmer, Wellesley, MA, USA). Each treatment was replicated three times.

### 2.7. Determination of Secondary Metabolite Content

Total flavonoid content was determined by the Al(NO_3_)_3_ color development method [34]. Total phenol content was determined by Folin–Ciocalteu method [35]. Total saponin content was determined by vanillin–perchloric acid color development method [36]. Cannabidiol (CBD) was extracted by ultrasound-assisted extraction and content determination by high performance liquid chromatography [37]. Each of the above had three replicates.

### 2.8. Statistical Analysis

Data from three biological replicates were analyzed using the Statistical Package for the Social Sciences (SPSS 21.0) (Chicago, IL, USA). Analysis of ANOVA (one-, two-, or three-way ANOVA) and the DunCan test were used to determine the differences between treatment groups (*p* < 0.05). All data in graphs and tables are raw data and are expressed as mean ± standard error (mean ± SE). *p* < 0.05 is considered a significant difference. Results were plotted using Origin 2021.

## 3. Results

### 3.1. AMF Colonization Rate and Mycorrhizal Dependence in Plant Roots

**Figure 1:**
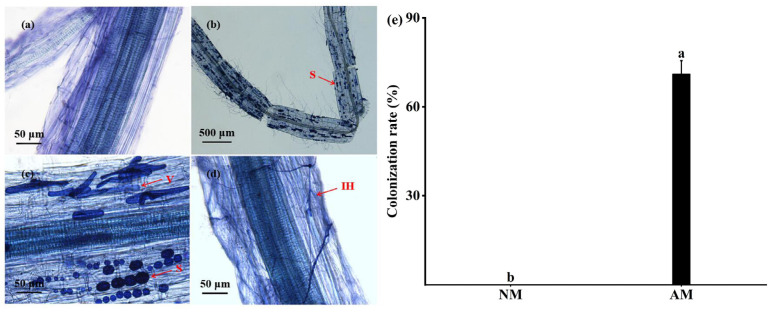
*F. mosseae* colonization of hemp roots and its effective colonization rate under a microscope before the start of treatment. Note: (**a**) NM-treated group; (**b**) spores (S) in the AM-treated group; (**c**) vesicles (V) in the AM-treated group; (**d**) intra-root hyphae (IH) in the AM-treated group; (**e**) AMF colonization rate of hemp root systems. Bar graphs represent means ± S.E. Different letters indicate significant differences between plants with and without AMF.

The *F. mosseae* established a symbiotic relationship with hemp before stress. Figure 1b–d shows clear spore, vesicle, and hyphae structures in hemp roots. However, fungal colonization was not detected in the NM treatment group, indicating the absence of native AMF in the soil matrix (Figure 1a). By determining the mycorrhizal colonization rate, it was found that *F. mosseae* colonization in hemp roots reached 71% before the start of the stress treatments (Figure 1e), which was significantly different from the NM treatments (Table S1). This study found that the CRAS of the CK was up to 89.99% (Table 2) and this was significantly different from the other treatment (Table S1). In addition, the CRAS under S2 was the lowest with only 72.69%; however, this was still higher than before stress. As the stress levels continued to increase, the CRAS decreased significantly (Table 2 and Table S1).

Two-way ANOVA indicated that the MD of hemp was affected by salt concentration and drought degree and the interaction of the two factors (Table S1). MD increased significantly with increasing salt concentration. In salt treatment, MD was 7.28%, 30.55%, and 59.27% for CK, S1 and S2, respectively. MD increased significantly with increasing drought. In the drought treatment, MD was 34.49% and 55.24% for D1 and D2, respectively. In salt–drought interaction stress, MD under S1D1 condition increased 4.49-fold compared to CK; however, the difference in MD with S1 and D1 was not significant.MD increased significantly with increasing levels of salt–drought interaction stress. Under the S1D2 condition, MD increased by 71.77% compared to the S1D1 (Table 2).

### 3.2. Growth Parameter

The growth parameters of hemp significantly decreased with increasing salt concentration or drought degree (Table S2). The reduction in growth parameters was more significant under salt–drought interaction stress than salt stress or drought stress alone. For example, plant DW was reduced by 35.14% and 39.64% under S1 and D1 treatments, respectively, relative to CK under uninoculated AMF conditions, whereas plant DW was reduced by 54.95% under S1D1 conditions. Inoculation with *F. mosseae* significantly increased the FW, DW, SD, NB, and RWC of plants in all treatments. For example, the FW of *F. mosseae* inoculated plants increased by 6.32%, 67.68%, and 110.4% relative to non-AMF-inoculated plants under CK, D1, and D2 conditions, respectively. However, the effect of *F. mosseae* inoculation on plant height was not significant. Three-way ANOVA indicated that plant height was only affected by drought degree (DD).The interaction of inoculated AMF with salt concentration (In *×* SC) and the interaction of inoculated AMF with drought degree (In *×* DD) did not affect SD. However, inoculated AMF (In) and the interaction of inoculated AMF, salt concentration, and drought degree (In *×* SC *×* DD) significantly affected plant SD (Table S1).

### 3.3. MDA, Proline, Soluble Sugar, and Soluble Protein Content

**Figure 2:**
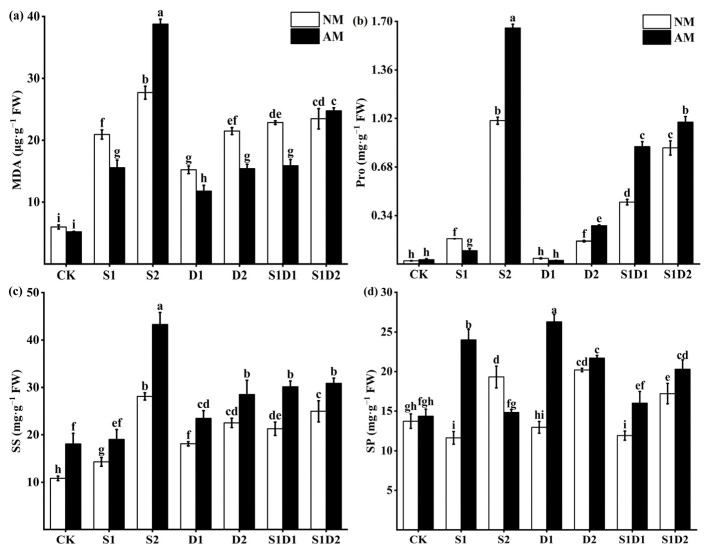
MDA (**a**), Pro (**b**), SS (**c**), and SP (**d**) content of hemp under salt and drought stress. Bars indicate mean ± S.E. Different letters indicate significant differences between hemp with and without AMF under each treatment.

Under non-inoculated AMF conditions, salt, drought, and salt–drought interaction stresses all resulted in increased MDA, Pro, and SS content in hemp, which increased with increasing levels of stress (Figure 2a–c). For example, the MDA content of hemp was 3.50 and 4.62 times higher in the S1 and S2 conditions than in the CK condition, respectively. In contrast, the Pro content of hemp was 2 and 8 times higher in the D1 and D2 conditions than in the CK condition, respectively. In addition, the SP content tended to decrease and then increase at increasing levels of stress (Figure 2d). For example, under S1 conditions, the SP content of hemp was reduced by 15.37% compared to CK. Whereas, under S2 conditions, the SP content of the plants increased by 40.71% compared to CK. The MDA, Pro, SS, and SP content under salt–drought interaction stress conditions exhibited similar trends with increasing stress levels as those of salt stress or drought stress alone (Figure 2a–d). For example, under S1D1 conditions, MDA content was 2.81-fold and 0.09-fold higher than CK and S1, respectively. Under S1D2 conditions, MDA content was 2.92-fold and 0.12-fold higher than CK and S1, respectively. In the case of *F. mosseae* inoculation, hemp exhibited a further increase in SS and SP content under salt, drought, or salt–drought interaction stress conditions, along with a decrease in MDA content (Figure 2a–d). For example, the SS content of hemp inoculated with *F. mosseae* was increased by 33% and 53.93% under S1 and S2 conditions, respectively, compared to that of hemp not inoculated with AMF.As the salt and drought stress levels increased, inoculation with *F. mosseae* resulted in a trend of decreasing and then increasing plant Pro content (Figure 2b). For example, inoculation with *F. mosseae* resulted in a 46.89% decrease in plant Pro content under S1 conditions compared to no AMF inoculation. In contrast, under S2 conditions, Pro content increased by 65%. In addition, Pro content was significantly increased in *F. mosseae*-inoculated plants under salt–drought interaction stress conditions. However, the effect of *F. mosseae* inoculation on plant MDA and SP under S2 conditions showed an opposite trend to that of the other treatments compared to that of the non-inoculated AMF. Specifically, MDA content increased by 39.98% and SP content decreased by 23.24% in *F. mosseae*-inoculated plants compared to non-AMF-inoculated plants under S2 conditions. Three-way ANOVA indicated that MDA content was not affected by inoculation with AMF (In), whereas SS was not affected by the interaction of inoculation with AMF and drought degree (In *×* DD), the interaction of salt concentration and drought degree (SC *×* DD), or the interaction of inoculation with AMF, salt concentration, and drought degree (In *×* SC *×* DD). Except for the above, all other treatments significantly affected the content of MDA, Pro, SS, and SP (Table S1).

### 3.4. SPAD Values and Photosynthesis Parameters

**Figure 3:**
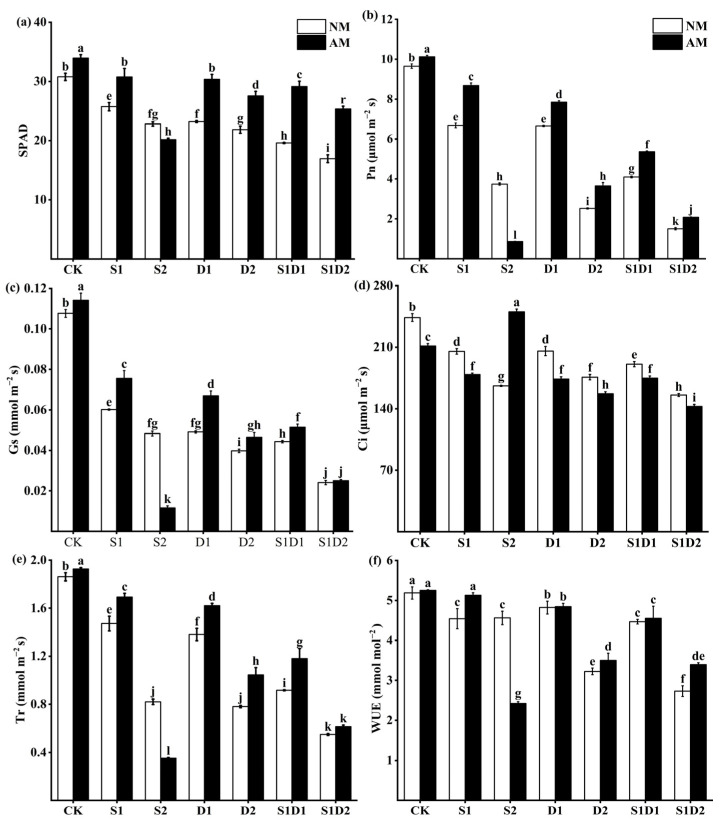
SPAD (**a**), Pn (**b**), Gs (**c**), Ci (**d**), Tr (**e**), and WUE (**f**) content of hemp under salt and drought stress. Bars indicate mean ± S.E. Different letters indicate significant differences between hemp with and without AMF under each treatment.

The study found that the photosynthetic parameters of hemp decreased significantly with increasing salt concentration or drought. The decrease in photosynthesis was more pronounced when the plants were subjected to both salt and drought stresses together. In fact, under the combined stress of salt and drought, the decrease in plant photosynthesis was much greater than under either salt or drought stress alone (Figure 3a–f). When the plants were not inoculated with AMF, their Pn decreased by 30.78% and 30.98% under S1 and D1 conditions, respectively, compared to the CK; however, under the combined stress of S1D1, Pn decreased by 57.62% compared to CK. On the other hand, when the plants were inoculated with *F. mosseae*, their SPAD, Pn, Gs, Tr, and WUE increased significantly under all treatment. In contrast, Ci decreased significantly. For instance, under CK, D1, and D2 conditions, the SPAD of inoculated *F. mosseae* plants was increased by 10.27%, 30.73%, and 26.29%, respectively, compared to non-inoculated plants. However, under S2 conditions, the effect of inoculation with *F. mosseae* on the photosynthetic parameters of the plants was opposite to that of the other treatments. Specifically, SPAD, Pn, Gs, Tr, and WUE decreased by 11.54%, 77.27%, 80%, 57.31%, and 46.93%, respectively, while Ci increased by 50.75% in AMF-inoculated plants compared to non-inoculated ones under S2 conditions. Three-way ANOVA indicated that the interaction of inoculated AMF, salt concentration and drought degree (In *×* SC *×* DD) had no significant effect on SPAD, Ci and WUE. Additionally, WUE was not affected by the interaction of salt concentration and drought degree (SC *×* DD). However, the interaction of inoculated AMF (In), salt concentration (SC), and drought degree (DD) significantly affected SPAD, Pn, Gs, Ci, Tr, and WUE (Table S1).

### 3.5. Fluorescence Parameters

**Figure 4:**
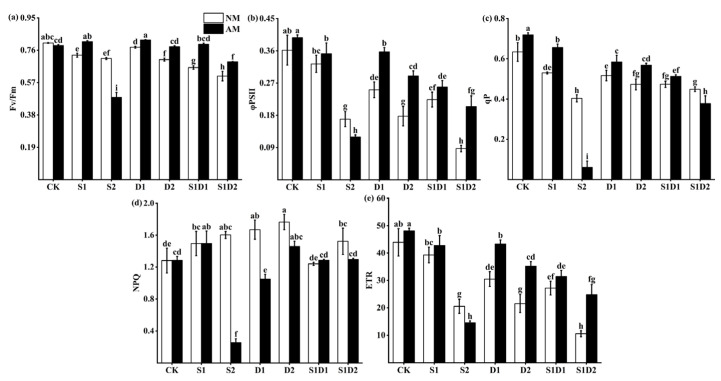
Fv/Fm (**a**), PS(II) (**b**), qP (**c**), NPQ (**d**), and ETR (**e**) content of hemp under salt and drought stresses. Bars indicate mean ± S.E. Different letters indicate significant differences between hemp with and without AMF under each treatment.

It was indicated that Fv/Fm, PS(II), qP, and ETR decreased significantly with increasing stress from Figure 4, while NPQ increased significantly (Figure 4a–e). The reductions in Fv/Fm, PS(II), qP, and ETR were more significant under salt–drought interaction stress than under salt or drought stress alone. For instance, the Fv/Fm of hemp leaves was reduced by 9.59% and 15.39% under S1D1 conditions compared to S1 and D1, respectively. The Fv/Fm of hemp leaves was reduced by 16.44% and 14.08% under S1D2 conditions compared to S1 and D2, respectively. Inoculation with *F. mosseae* resulted in a significant increase in plant Fv/Fm, PS(II), qP, and ETR, while decreasing NPQ. However, it is worth noting that the effect of *F. mosseae* inoculation on the fluorescence parameters of the plants under S2 conditions differed significantly from those of the other treatments, in which NPQ was further reduced. For example, under S2 conditions, *F. mosseae* inoculation resulted in a 32.39%, 29.41%, 85%, and 29.36% decrease in Fv/Fm, PS(II), qP, and ETR, respectively, in hemp compared to uninoculated AMF plants. In addition, NPQ decreased by 82.5% in the hemp inoculated with *F. mosseae* compared to the plants that were not inoculated. Three-way ANOVA indicated that the interaction of salt concentration and drought degree (SC *×* DD) with inoculation of AMF, salt concentration, and drought degree (In *×* SC *×* DD) had no significant effect on PS(II) and ETR of plants. The interaction of salt concentration and drought degree (SC *×* DD) did not significantly affect the qP of the plants. Inoculation with AMF (In) had no significant effect on Fv/Fm. Furthermore, the interaction of inoculation with AMF (In), salt concentration (SC), and drought degree (DD) significantly affected the fluorescence parameters of the plants (Table S1).

### 3.6. Ionic Content

**Figure 5:**
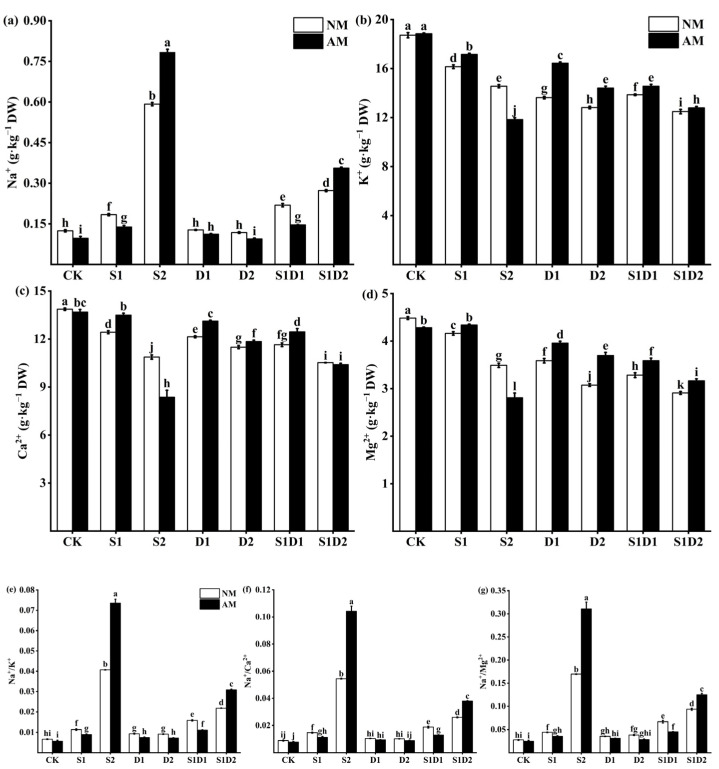
Na^+^ (**a**), K^+^ (**b**), Ca^2+^ (**c**), Mg^2+^ (**d**), Na^+^/K^+^ (**e**), Na^+^/Ca^2+^ (**f**), and Na^+^/Mg^2+^ (**g**) content of hemp leaves under salt and drought stress. Bars indicate mean ± S.E. Different letters indicate significant differences between hemp with and without AMF under each treatment.

In the study, it was found that inoculation with *F. mosseae* had a significant impact on the ion content in hemp leaves under different stress conditions. The content of K^+^, Ca^2+^, and Mg^2+^ in the plant showed a decrease with increase in stress levels (Figure 5b–d). For instance, under S1 and S2 conditions, K^+^ levels decreased by 13.73% and 22.22%, respectively, when compared to CK. On the other hand, the Na^+^ content in hemp leaves increased with an increase in salt concentration, and the interaction of salt concentration and drought caused Na^+^/K^+^, Na^+^/Ca^2+^, and Na^+^/Mg^2+^ ratios to increase (Figure 5a,e–g). For example, Na^+^ levels increased by 50% and 3.92-fold under S1 and S2 conditions, respectively, compared to CK, and the corresponding Na^+^/K^+^, Na^+^/Ca^2+^, and Na^+^/Mg^2+^ ratios also saw an increase. However, drought stress had no significant impact on the Na^+^ content of the plants. Additionally, drought stress increased the Na^+^/K^+^, Na^+^/Ca^2+^, and Na^+^/Mg^2+^ ratios. Nonetheless, the changes in drought degree did not have a significant effect on Na^+^/K^+^ and Na^+^/Ca^2+^ ratios. For instance, Na^+^/K^+^ increased by 40.96% and 38.4% under D1 and D2 conditions, respectively, compared to CK. Inoculation with *F. mosseae* regulated of Na^+^ content, Na^+^/K^+^, Na^+^/Ca^2+^, and Na^+^/Mg^2+^ ratios under stress conditions. However, the results of *F. mosseae* inoculation under S2 and S1D2 conditions were opposite to the other treatments. For example, the Na^+^ content, as well as Na^+^/K^+^, Na^+^/Ca^2+^, and Na^+^/Mg^2+^ ratios were reduced by 91.67%, 21.42%, 0.34%, and 19.89%, respectively, in plants inoculated with *F. mosseae* compared to those that were not inoculated with AMF under S1. In S2, the Na^+^ content, as well as the Na^+^/K^+^, Na^+^/Ca^2+^, and Na^+^/Mg^2+^ content, were increased by 47.46%, 80.92%, 91.35%, and 83.02%, respectively, in plants inoculated *F. mosseae* plants compared to those that were not inoculated with AMF. In addition, inoculation with *F. mosseae* increased the K^+^, Ca^2+^, and Mg^2+^ content in the stress conditions. However, Ca^2+^ and Mg^2+^ content was significantly reduced under CK conditions. K^+^, Ca^2+^, and Mg^2+^ content also tended to decrease under S2 conditions. For instance, K^+^, Ca^2+^, and Mg^2+^ increased by 20.62%, 8.07%, and 10.31%, respectively, in plants inoculated with *F. mosseae* plants compared to those that were not inoculated with AMF under D1 conditions. Under CK conditions, Ca^2+^ and Mg^2+^ content decreased by 1.23% and 4.46%, respectively, in plants inoculated with *F. mosseae* compared to those that were not inoculated with AMF. Under S2 conditions, K^+^, Ca^2+^, and Mg^2+^ were reduced by 18.68%, 23%, and 19.48%, respectively, in plants inoculated with *F. mosseae* compared to those that were not inoculated with AMF. Three-way ANOVA showed that all treatments significantly affected Na^+^, K^+^, Ca^2+^, and Mg^2+^ content as well as Na^+^/K^+^, Na^+^/Ca^2+^, and Na^+^/Mg^2+^ ratios in hemp leaves (Table S1).

### 3.7. Secondary Metabolite Content

**Figure 6:**
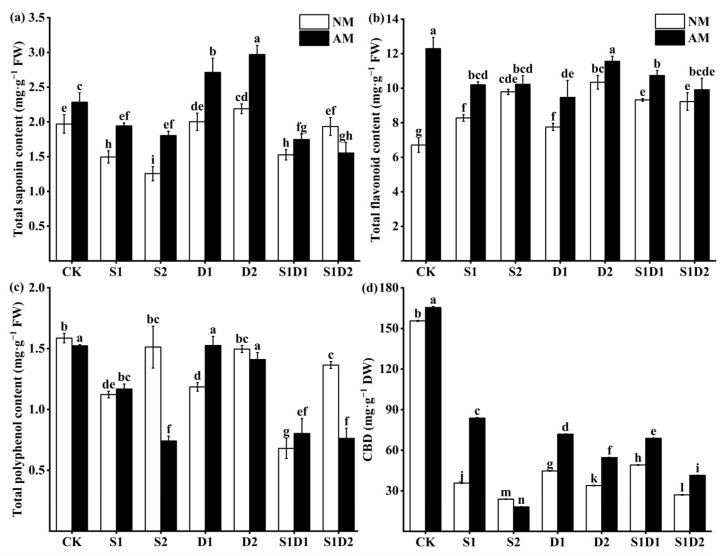
Total saponins (**a**), total flavonoids (**b**), total phenols (**c**), and CBD (**d**) content of hemp leaves under salt and drought stress. Bars indicate mean ± S.E. Different letters indicate significant differences between hemp with and without AMF under each treatment.

The content of total saponin in hemp leaves decreased significantly with the increase in salt concentration (Figure 6a). In plants not inoculated with AMF, the total saponin content in hemp leaves was 23.86% and 36.04% lower in the S1 and S2 treatments, respectively, compared to the CK. On the other hand, the total saponin content increased with increasing drought. In the plants not inoculated with AMF, the total saponin content of hemp leaves increased by 1.52% and 11.17% in the D1 and D2 treatments, respectively, when compared to the CK. The total saponin content of hemp leaves decreased and then increased as the interaction of salt concentration and drought were enhanced. In the plants not inoculated with AMF, the total saponin content of hemp leaves under the S1D1 and S1D2 treatments decreased by 22.34% and 2.03%, respectively, compared to CK. The study also found that the total flavonoid content in hemp leaves increased, and that CBD content decreased significantly with increasing stress levels (Figure 6b,d). For instance, in the absence of AMF, the total flavonoid content of hemp leaves increased by 23.4% and 45.9%, under the S1 and S2 treatments, respectively, while the CBD content decreased by 76.95% and 84.69%, respectively, compared to the CK. Moreover, the total phenolic content of hemp leaves showed a decrease and then an increase with increasing stress levels (Figure 6c). For example, in the absence of AMF, the total phenolic content of hemp leaves under D1 and D2 treatments decreased by 25.16% and 5.66%, respectively, compared to the CK. Inoculation with *F. mosseae* increased the content of total saponins under salt stress, drought stress, and S1D1 treatments, However, it decreased the content of total saponins under S1D2 treatment. For instance, the total saponin content of *F. mosseae*-inoculated plants under S1D1 treatment increased by 14.38% compared to those not inoculated with AMF. However, the content of total saponins of plants under S1D2 condition decreased by 19.69%. Additionally, inoculation with *F. mosseae* increased the total flavonoid content under stress conditions. When inoculating plants with *F. mosseae*, it was found that the total flavonoid content increased by 23.19% and 22.04% under S1 and D1 conditions, respectively, compared to not inoculated ones. In contrast, inoculation with *F. mosseae* led to a decrease in CBD content under the S2 condition, but an increase in CBD content under other treatments. For example, *F. mosseae*-inoculated plants grown under S1 conditions had 1.34 times higher CBD content than non-inoculated plants, while those grown under S2 conditions had 23.8% lower CBD content. Furthermore, the total phenol content of *F. mosseae*-inoculated plants increased by 30.36%, 60.5%, and 47.06% under S1, D1, and S1D1 conditions, respectively, compared to non-inoculated plants. In contrast, the total phenol content of *F. mosseae*-inoculated plants decreased by 39.07%, 17.33%, and 30.66% under S2, D2, and S1D2 conditions, respectively. Three-way ANOVA indicated that inoculation with AMF (In) had no significant effect on total phenol content. All the treatments, except for the above, significantly altered the total saponin, total flavonoid, total phenol, and CBD content of the plants (Table S1).

### 3.8. Heat Map Analysis

**Figure 7:**
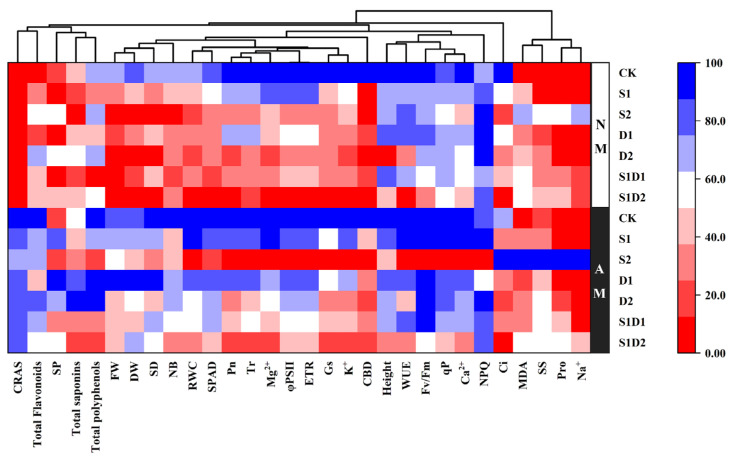
Heat map analysis of hemp inoculated with AMF, salt stress, and drought stress treatments. In the heat map, the color gradient sets bright red as the minimum, dark blue as the maximum, and white as the middle, with an asymptote (or gradient) between these endpoints.

The heat map depicted in Figure 7 shows the relative performance of treated plants under salt stress and drought stress conditions. The dark blue color indicates the highest values, while the bright red color represents the lowest values (Figure 7). Under salt stress conditions, FW, DW, SD, NB, RWC, SPAD, Ci, K^+^, and CBD showed significant differences compared to CK, with the values being higher under CK conditions. Under drought stress conditions, DW, SPAD, Pn, Gs, Ci, Tr, φPS(II), ETR, K^+^, Mg^2+^, and CBD were also significantly different compared to CK, with the values being higher under CK conditions. Salt–drought interaction stress conditions showed significant differences in all parameters except for soluble substances, NPQ, Na^+^, CRAS, total saponin, total flavonoids, and total phenols compared to CK, with the values being higher under CK conditions. *F. mosseae* colonization significantly increased CRAS in the presence or absence of stress. Inoculation with *F. mosseae* shows a positive effect on the synthesis of secondary metabolites, such as total flavonoids and total phenols, under CK and drought conditions. Under drought conditions, *F. mosseae* inoculation also showed a positive effect on total saponin, total flavonoids, and total phenols. Under the D1 condition, the growth and development of hemp (FW, DW, SD, and NB) were found to be correlated with *F. mosseae* inoculation, with higher values observed with *F. mosseae* inoculation. Under S2 conditions, *F. mosseae* inoculation significantly increased the MDA, Pro, SS, and Na^+^ values of the plants. However, except for S2, the effect of *F. mosseae* inoculation on the MDA, Pro, SS, and Na^+^ of the plants was only moderately strong. Inoculation with *F. mosseae* also enhanced the growth (FW, DW, SD, NB, RWC) and PS(II) activity (φPS(II), ETR, Fv/Fm) of hemp under salt–drought interaction stress conditions. However, as the degree of salt–drought interaction stress increased, the effect of *F. mosseae* inoculation on them subsequently weakened. This suggests that AMF inoculation can positively affect the growth of hemp under lighter salt–drought interaction stress conditions.

## 4. Discussion

AMF can help increase host plant’s resistance to various stresses, such as salinity, drought, heavy metals, and temperature extremes [38]. Studies show that AMF contributes to mitigating oxidative stress damage in host plants during adversity stress conditions [12], regulating their soluble substances [39] and metal elements content [40], enhancing gas exchange capacity [2], and increasing the production of secondary metabolite [41], among other benefits. AMF achieves these effects by forming specialized structures in association with the roots of host plant. These structures include the extraradical structure, which consists of extraradical hyphae and spores in the soil, as well as the intraradical structure, which consists of arbuscule, intraradical hyphae, and vesicles [42]. The extra- and intra-root hyphae are responsible for nutrient uptake from the soil and nutrient transport, respectively [43,44]. Spores, on the other hand, serve as storage structures for asexual reproductive organs and lipids. The arbuscular are responsible for the exchange of material between the fungus and the plant. The vesicles are responsible for storing nutrients and reproducing subsequent generations [42].

In this study, it was found that hemp was able to establish a good symbiotic with *F. mosseae*. The CRAS decreased with increasing salt concentration, drought degree, and salt–drought interaction stress. This suggests the negative effect of stresses on mycelial growth [9,45]. However, CRAS was lowest in S2 condition. This indicated that high salt stress had the most significant inhibitory effect on mycelial growth. The contribution of AMF to the host plant biomass can be reflected by its MD. When grown under CK condition, the MD of hemp was only 7.28%. This may be attributed to the characteristics of hemp, which is a high biomass plant with a broad root system that is highly efficient in terms of nutrient uptake [46]. This leads to a relatively low dependence of hemp on AMF under normal conditions. However, as stress levels increase, the MD also increases, which is consistent with the results obtained by Zhang et al. [47] when studying the effect of AMF on maize under molybdenum toxicity.

Plant growth and development are inhibited under salt, drought, or salt–drought interaction stress conditions. However, AMF was able to improve the inhibitory effects of those stresses on host plants [38,48], which is consistent with the results of this study. This study found that salt, drought, or salt–drought interaction stresses significantly reduced the FW, DW, SD, NB, and RWC of hemp. However, *F. mosseae* inoculation mitigated the negative effects of these stresses on plant growth. The mycorrhizal symbiosis created by AMF with the host plant root assisted the plant in absorbing more water and nutrients, which in return facilitated the plant’s material accumulation [49]. In this study, the effects of stress and *F. mosseae* inoculation on hemp plant height were not significant. Liu et al. [50] divided the whole life cycle of hemp into four stages, namely the seedling stage, seedling growth stage, rapid growth stage and mature stage; the seedling to rapid growth stage lasted about 95–105 days, and the growth in hemp plant height was the most significant during this period. In the experimental method of this study, the whole process consisted of three stages: seedling, *F. mosseae* inoculation, and stress, lasting 7, 55, and 23 days, respectively. Among them, the seedling and *F. mosseae* inoculation stages occupied 72.94% of the whole process. This longer period of time may be a crucial reason for the insignificant changes observed in the hemp plant height after stress. Additionally, the RWC of *F. mosseae* inoculated plants was significantly lower than that of plants not inoculated with AMF under S2 conditions. This is because AMF obtains carbon sources from host plants to support their own growth and development, energy consumption and building cellular carbon skeletons. In return, AMF helps host plants to absorb water and mineral nutrients and helps to increase plant resistance [51]. However, under extreme stress conditions, the symbiotic balance between AMF and the host plant is disrupted, resulting in the net cost of AMF binding to hemp exceeding the net benefit [46,52]. In other words, AMF dominates the host plant in the competition for carbon-source distribution, which ultimately results in the reduced RWC of the host plant. This was supported by the changes in physiological indicators and secondary metabolite content of hemp inoculated with *F. mosseae* under S2 conditions. However, other growth indicators of hemp inoculated with *F. mosseae* under S2 conditions were significantly higher than those of the treatment not inoculated with AMF, which was due to the accumulation of substances prior to stress treatments. The data showed that the salt–drought interaction stress had a more significant inhibitory effect on plant growth than a single stress. Meanwhile, inoculation with AMF was able to alleviate the effect of salt–drought interaction stress on plant growth, which is consistent with the results of Wang et al. [48].

Adverse stress leads to elevated levels of reactive oxygen species (ROS) in plants, which can damage plant cell membranes [9]. The peroxidation of lipid membranes can be measured by MDA which is used to assess the degree of damage to the plant cells [53,54]. The data revealed that salt, drought stress, and salt–drought interaction stress caused a significant increase in MDA content, and the degree of damage increased with the level of stress, which is consistent with the results obtained by Luo et al. [55] when studying the effect of AMF on *Dysosma versipellis* under copper stress. Except for the S2 and S1D2 conditions, inoculation with *F. mosseae* was able to significantly reduce the MDA content. This implies that AMF can mitigate the oxidative damage caused by stress, which is consistent with the results obtained by Li et al. [56] when studying the effect of AMF on *Medicago truncatula* under cadmium stress. Under S2 and S1D2 conditions, inoculation with *F. mosseae* led to an increase in MDA content, indicating that the symbiotic balance between AMF and the host plant was disrupted under extreme stress. This resulted in competition between AMF and the host plant for the C source, aggravating the stress on the plant and ultimately leading to an increase in MDA content. The changes in MDA content under S1D2 conditions suggested that the competition between AMF and the host plant increased with an increase in the stress level.

Plants can regulate the osmotic balance of their cells by producing osmoregulatory substances, which help them become more resistant to stress [57]. In the present study, it was observed that as stress levels increased the Pro content in plan also increased, which indicates that the plants enhanced their stress tolerance by producing more the Pro content. When hemp was inoculated with *F. mosseae* under salt or drought stress conditions, the content of Pro tended to decrease and then increase as the stress level increased. AMF can increase plant resistance, which means that under mild stress conditions, plants don’t need to rely on producing more Pro content to regulate plant resistance. However, as the stress level increases, the plant is stimulated to increase the Pro content, which ultimately increases plant resistance. The inoculation of *F. mosseae* significantly increased the content of Pro in plants under salt–drought interaction stress conditions, which is consistent with the results obtained by Wang et al. [48] when studying the effect of AMF on *Leymus chinensis* under alkali and drought stresses. This suggested that AMF can enhance plant performance under adverse stress by regulating Pro levels in the host plant. Studies by Zong et al. [40] and Carrara et al. [14] indicated that inoculation with AMF can also increase a plant’s SS and SP content, which is consistent with the results of this study. However, under S2 conditions, inoculation with *F. mosseae* reduced plant SP content compared to non-inoculated plants. This result indicated that under S2 conditions, AMF-inoculated hemp is more susceptible to stress damage. In summary, high salt stress (S2) and AMF inoculation together caused damage to plants.

The SPAD values are a measure of the relative chlorophyll content of plant leaves, indicating the plant’s health status [58]. The study indicated that adverse stress can lead to a decrease in chlorophyll content, resulting in low SPAD values [59]. In contrast, inoculation with *F. mosseae* significantly increases the SPAD values of host plants [60], which is consistent with the effect of AMF on the SPAD values of hemp in the present study. However, in this study, *F. mosseae* inoculation significantly reduced SPAD values in host plant leaves compared to in non-inoculated plants under S2 conditions. This reduction is consistent with previous research showing that AMF inoculation to enhance hemp and cope with high salt stress may cause stress damage. Photosynthesis is a key physiological process affecting plant growth. In this study, the photosynthetic indexes (Pn, Tr, Ci, Gs, and WUE) of hemp gradually decreased as stress levels increased, which is consistent with the results obtained by Zong et al. [40] when studying the effect of AMF on *Xanthoceras sorbifolium* under salt stress. Studies have shown that inoculation with AMF increases the photosynthetic capacity of the plant [61]. The research results of Begum et al. [11] and Zhang et al. [9] indicated that the photosynthetic capacity of leaves of AMF-inoculated plants was higher than that of plants not inoculated with AMF under drought or salt stress conditions. In this study, except for in the S2 condition, the Pn, Tr, Gs, and WUE of *F. mosseae* inoculated plants were significantly higher than those of uninoculated plants, while Ci was significantly lower. This indicates that AMF improved the gas exchange capacity and enhanced the photosynthetic performance of the plants. Thus, AMF inoculation increases plant resistance to adversity stress by regulating the gas exchange properties of plants [9]. Notably, the effects of AMF on hemp Pn, Tr, Gs, WUE, and Ci under S2 conditions were opposite to the previous results. This is consistent with the changes in the results, such as the RWC of AMF-inoculated hemp under S2 conditions. Therefore, it can be concluded that high salt stress and AMF inoculation together caused damage to the plant, resulting in reduced gas exchange capacity.

Chlorophyll fluorescence parameters play a crucial role in evaluating plant responses to ecophysiological stress [62,63,64,65,66]. Plants maintain the balance between photosynthetic electron transport and carbon metabolism through nonphotochemical processes, and by increasing the electron transport activity of PSII [67]. In the present study, Fv/Fm, PS(II), qP and ETR decreased significantly with increasing stress level, while NPQ increased significantly. A decrease in Fv/Fm indicates damage to the PS II system. Decrease in ΦPS II indicates that the formation of assimilative forces, such as ATP and NADPH, is blocked. Decrease in qP indicates that electron transfer to the PS II reaction centers is inhibited. Decrease in ETR indicates that the plant is reducing the efficiency of photosynthesis and that the rate of synthesis of photosynthates is slowing down. On the other hand, the rise in NPQ indicates that the plant is actively improving its ability to dissipate heat in order to consume excess excitation energy, which can protect photosynthetic pigments from excess light and mitigate the damage caused by ecophysiological stress [68]. Inoculation with AMF increases the photochemical activity of PSII reaction centers, improves heat dissipation rates, and reduces damage to PSII photosynthetic centers by abiotic stresses. As a result, it reduce inhibition of photosynthetic electron transport [69]. In the present study, plants inoculated with *F. mosseae* showed a significant increase in Fv/Fm, PS(II), qP, and ETR when subjected to salt, drought, or salt–drought interaction stress conditions, except for the S2 condition. This suggests that these types of stress inhibits chlorophyll synthesis and damage the structure of plant chloroplasts. However, inoculation with AMF can alleviate chlorophyll degradation and chloroplast disintegration to a certain extent, improving the electron transfer activity of PSII and increasing plant stress tolerance, which is consistent with the results obtained by Li et al. [70] when studying the effect of AMF on *Antirrhinum majus* L. under low-temperature and weak-light conditions. Under S2 conditions, *F. mosseae* inoculation had the opposite effect, reducing Fv/Fm, PS(II), qP, and ETR, and NPQ was significantly lower than the normal level in stressed plants compared to those not inoculated with AMF. This may be due to the fact that inoculation of AMF under high salt stress caused more damage to the chlorophyll and chloroplast, disrupting the balance between photosynthetic electron transfer and carbon metabolism, and leading to a decrease in the electron transfer activity of PSII. Notably, under S1D2 conditions, the qP of the plants inoculated with *F. mosseae* was significantly decreased compared to those not inoculated with AMF, but not as significantly as that of the plants inoculated with *F. mosseae* under S2. This indicates that electron transfer to the PSII reaction centers was inhibited, but to a lesser extent compared to S2. This may be due to the increased competition between AMF and host plants caused by the elevated stress level. In this study, NPQ decreased in plants inoculated with *F. mosseae* under D1, D2, and S1D2 conditions. This may be due to the fact that abiotic stress affects the photosynthetic properties of leaves, altering the distribution of chlorophyll fluorescence, and resulting in a decrease in the openness of PSII reaction centers and a weakening of photosynthetic electron transport activity [71].

Plant sensitivity to salt mainly depends on the uptake, accumulation, and distribution of Na^+^. Increased levels of salt and salt–drought interaction stresses result in a higher Na^+^ content but drought stress does not significantly affect Na^+^ accumulation. In the present study, inoculation with *F. mosseae* reduced Na^+^ content in hemp leaves, except for under the S2 and S1D2 conditions. There are two possible explanations for this phenomenon. First, plants inoculated with AMF may limit the transfer of toxic ions from the roots to aboveground by retaining Na^+^ in intraradical hyphae and vesicles [72]. Second, AM may induce the expression of Na^+^/H^+^ transporters that help plants store Na^+^ in the vesicles, preventing it from entering the roots [73]. However, under the S2 and S1D2 conditions, the Na^+^ content of hemp plants inoculated with *F. mosseae* was different from the other treatment groups. This suggest that AMF inoculation may be effective in reducing the accumulation of toxic ions in plant photosynthetic tissues, which is consistent with the results obtained by Porcel et al. [74] when studying the effect of AMF on rice plants under salt stress. However, high salt stress can upset the symbiotic balance between AMF and plants, leading to further exacerbation of plant stress. This means that there is a limit to how much AMF can help plants to adapt to high levels of stress. Interestingly, in the S2 treatment, inoculation with *F. mosseae* resulted in significantly higher Na^+^ content in hemp leaves compared to when there was no AMF inoculation. This result suggests that the Na^+^ accumulation in plant leaves caused by high salt stress and inoculation with AMF may be one of the main factors leading to plant damage. Potassium, calcium, and magnesium are essential mineral elements for plant growth, and they play crucial roles in maintaining osmotic regulation, promoting photosynthesis, and facilitating nutrient uptake and transport. The balance of intracellular ion concentrations is essential for the metabolism of living cells [48]. To survive, plant cells need to maintain low concentrations of toxic ions and accumulate essential ions by regulating ion fluxes appropriately [75]. In this study, stress caused a decrease in K^+^, Ca^2+^, and Mg^2+^ content in hemp leaves, and the higher the level of stress, the lower the content. However, inoculation with *F. mosseae* increased levels of K^+^, Ca^2+^, and Mg^2+^ content, but, under high salt conditions, it led to a decrease in K^+^ content instead. Inoculation with *F. mosseae* increased the levels of K^+^, Ca^2+^, and Mg^2+^ in hemp and maintained high levels of Na^+^/K^+^, Na^+^/Ca^2+^ and Na^+^/Mg^2+^. The data indicates that inoculation with AMF promoted the accumulation of K^+^, Ca^2+^, and Mg^2+^ in plants. The reason behind this could be that the mycorrhizal symbiosis increased the length of roots and root area through the action of hyphae and arbuscular, thereby effectively increasing the ability of plants to absorb mineral nutrients. On the other hand, K^+^, Ca^2+^, and Mg^2+^, as osmotic substances, can be selectively absorbed by the mycorrhizal symbiosis and transported to plant organs and tissues to prevent the plant from absorbing more Na^+^ [76]. The results showed that inoculation of AMF could regulate plant ionic homeostasis and help hemp adapt to different concentrations of salt stress, which is consistent with the results obtained by Zhang et al. [9] when studying the effect of AMF on *Oryza sativa* L. plants under salt stress.

Plant adaptation to complicated environmental stresses involves important factors, such as total saponins, total flavonoids, and total phenols. These compounds have multiple roles in plant under adverse stress, including antioxidant functions, regulation of growth and development, enhancement of water use efficiency, maintenance of cell membrane stability, involvement in signaling pathways, and expression of resistance genes [77,78,79,80]. Some studies have investigated the effect of drought stress on these secondary metabolites of *Agave salmiana*. The results showed that drought stress tends to increase total saponin content, while total phenol content shows an initial decrease followed by an increase. Salt stress, on the other hand, decreases total saponin and total phenol content while increasing flavonoid content in plants [81,82,83,84]. These findings are consistent with the effect of AMF on the secondary metabolite content of hemp under salt and drought stress in the present study. Inoculation with AMF has been shown to increase plant secondary metabolites under adverse stresses [85,86]. Specifically, inoculation with *F. mosseae* significantly increased the content of total saponins, total flavonoids, and total polyphenols in hemp under salt, drought, or salt–drought interaction stress. However, under salt–drought interaction stress, inoculation with *F. mosseae* led to a significant decrease in total saponin and total phenol content in the plants compared to those not inoculated with AMF, this reduction may be attributed to the combined effect of salt–drought interaction stress. The highest level of total flavonoid content was observed in the plants inoculated with *F. mosseae* under stress-free conditions, indicating that AMF could promote total flavonoid synthesis in plants. Under S2 conditions, the total phenol content of plants inoculated with *F. mosseae* was significantly lower than that of non-inoculated AMF plants. This reduction could be attributed to the combination of high salt stress and the fact that inoculation with AMF can cause damage to the plants, leading to a decrease in the total polyphenol content. Recent studies have highlighted that the addition of tea polyphenols [87] and muscimol [88,89] can help protect against adverse stress by increasing plant antioxidant activity and scavenging reactive oxygen species. CBD is a unique secondary metabolite found in industrial cannabis flowers and leaf organs, with a variety of bioactivities, including anti-inflammatory, antioxidant, antimicrobial, and neuroprotective properties [90,91]. However, the CBD content was significantly reduced with the increase in stress level, possibly due to the accumulation of ROS in the plants, causing oxidative damage and inhibiting CBD production. Seemakram et al. [92] showed that inoculation with *Rhizophagus prolifer* PC2-2 and *R. aggregatus* BM-3g3 increased the CBD content in hemp by 29.07% and 31.43%, respectively, as compared to non-inoculated plants. In this study, inoculation with AMF also increased CBD, except under S2 conditions. This increase may be attributed to AMF improving the antioxidant capacity of the plant, promoting CBD accumulation and enhancing the host plant’s resistance. Under S2 conditions, the CDB content was found to be significantly reduced in plants that were inoculated with *F. mosseae*, as compared to non-inoculated plants. This reduction in CBD content may have occurred because the combination of high salt stress and AMF inoculation caused damage to the plant, which further decreased the CBD content. However, AMF can prove to be an effective inoculants to increase the content of plant secondary metabolites, especially under adverse stress. Under no-stress conditions, inoculation with AMF resulted in a significant increase in the CBD content of hemp. Therefore, using AMF as an inoculants may be an effective means of increasing CBD content in hemp.

## 5. Conclusions

Inoculation with AMF can help reduce the damage caused by salt, drought, and salt–drought interaction stresses in hemp plants. However, it can also exacerbate the damage caused by high salt stress. In addition to promoting plant growth, inoculation with AMF can also reduce the damage to lipid membrane peroxidation and increase the levels of osmoregulatory substances, K^+^, Ca^2+^, and Mg^2+^, while decreasing the levels of Na^+^ under salt and salt–drought interaction stress conditions. AMF can also improve gas exchange efficiency and PSII activity, as well as increase the content of secondary metabolites. However, it has the opposite effect on plants under high salt stress. Overall, the results suggest that AMF inoculation can be used as an effective way to reduce plant stress under salt, drought, and salt–drought interaction stresses. Additionally, it has the potential to increase the content of secondary metabolites under stress conditions, indicating potential application in the future.

## Figures and Tables

**Table 1:** Experimental treatment scheme.

Groups	Treatment Group	Methods
Control group	CK	45% soil moisture content
Salt stress group	S1	100 mM NaCl treatment
	S2	200 mM NaCl treatment
Drought stress group	D1	25% soil moisture content
	D2	15% soil moisture content
Salt–drought interaction stress group	S1D1	100 mM NaCl and 25% soil moisture
	S1D2	content co-treatment
Inoculation group	AM	Inoculation with AMF
Non-inoculated group	NM	No AMF inoculation

**Table 2:** Effective colonization rate of the hemp root and its MD under salt stress and drought stress.

Treatment Group	CRAS		MD
	NM	AM	
CK	0 ± 0 g	89.99 ± 1.67 a	6.1 ± 1.04 c
S1	0 ± 0 g	82.04 ± 1.81 c	31.53 ± 3.77 b
S2	0 ± 0 g	72.69 ± 0.88 f	57.92 ± 2.17 a
D1	0 ± 0 g	84.57 ± 1.28 b	34.49 ± 1.87 b
D2	0 ± 0 g	75.81 ± 1.27 e	55.24 ± 2.16 a
S1D1	0 ± 0 g	79.4 ± 1.14 d	33.47 ± 2.84 b
S1D2	0 ± 0 g	75.32 ± 1.04 e	57.49 ± 2.11 a

Values in the table represent mean ± S.E. Different letters indicate significant differences between indicators under each treatment.

## Data Availability

The data presented in this study are available on request from the corresponding authors. The data are not publicly available due to privacy.

## Supplementary Materials

The following supporting information can be downloaded at: https://www.mdpi.com/article/10.3390/jof10040283/s1, Table S1: Multifactorial analysis of variance (ANOVA) of the effects of inoculation (In), salt concentration (SC), and degree of drought (DD), and their interactions on physiological and biochemical parameters and secondary metabolites of hemp. Table S2: Growth parameters of hemp under salt stress and drought stress.
