# Supplementary material for: Arbuscular Mycorrhizal Fungi-Mediated Modulation of Physiological, Biochemical, and Secondary Metabolite Responses in Hemp (Cannabis sativa L.) under Salt and Drought Stress
Source: J Fungi (Basel). 2024 Apr 12;10(4):283. doi: 10.3390/jof10040283 (PMC11050865; doi:10.3390/jof10040283)
Supplement: Supplementary file 1 [file jof-10-00283-s001.zip › jof-2933154-supplementary.pdf]

Table S1. Multifactorial analysis of variance (ANOVA) of the effects of inoculation (In), salt concentration (SC) and degree of drought (DD) and their interactions on physiological and biochemical parameters and secondary metabolites of hemp

Dependent variable	Independent variable						
	In	*SC*	*DD*	*In×SC*	*In×DD*	*SC×DD*	*In×SC×DD*
Colonization rate before stress treatment	720.143 ***	-	-	-	-	-	-
Colonization rate under salt stress and drought stress	64854.825 ***	125.894 ***	93.492 ***	125.894 ***	93.492 ***	11.946 ***	11.946 ***
MD	-	293.922 ***	368.844 ***	-	-	53.701 ***	-
FW	2440.350 ***	620.480 ***	860.115 ***	68.303 ***	69.773 ***	59.340 ***	36.581 ***
DW	2364.265 ***	951.587 ***	948.884 ***	68.009 ***	94.324 ***	30.274 ***	51.733 ***
Height	0.011 ns	2.576 ns	9.901 ***	2.371 ns	0.783 ns	0.530 ns	2.450 ns
SD	278.388 ***	121.206 ***	120.313 ***	0.166 ns	2.031 ns	22.047 ***	7.741 **
NB	62.438 ***	59.500 ***	19.289 ***	7.622 **	3.267 ns	9.800 ***	2.022 ns
RWC	58.073 ***	121.907 ***	81.437 ***	22.211 ***	0.662 ns	0.695 ns	2.942 ns
MDA	0.855 ns	1690.603 ***	370.505 ***	141.183 ***	8.989 ***	67.203 ***	46.267 ***
Pro	1097.448 ***	7893.807 ***	1326.513 ***	491.128 ***	82.297 ***	561.527 ***	91.505 ***
SS	234.740 ***	300.804 ***	137.608 ***	14.847 ***	0.474 ns	2.405 ns	2.335 ns
SP	110.746 ***	11.503 ***	53.662 ***	65.952 ***	33.691 ***	71.889 ***	86.984 ***
SPAD	451.278 ***	437.212 ***	355.435 ***	60.502 ***	30.314 ***	4.892 *	0.288 ns
Pn	39.244 ***	15679.021 ***	18604.321 ***	1323.759 ***	21.684 ***	187.719 ***	132.571 ***

Gs	83.668 ***	2699.321 ***	1945.170 ***	298.675 ***	116.895 ***	212.269 ***	49.478 ***
Tr	11.192 **	2239.073 ***	2030.770 ***	128.726 ***	6.841 **	11.998 ***	16.808 ***
Ci	40.870 ***	260.051 ***	868.822 ***	305.710 ***	14.872 ***	67.730 ***	2.691 ns
WUE	34.580 ***	239.362 ***	509.860 ***	144.857 ***	6.113 **	0.253 ns	1.867 ns
Fv/Fm	0.141 ns	524.566 ***	113.776 ***	168.154 ***	56.047 ***	4.278 *	11.221 ***
PS(Ⅱ)	47.700 ***	220.083 ***	171.057 ***	7.892 **	10.390 ***	3.287 ns	2.482 ns
qP	28.563 ***	618.246 ***	151.960 ***	179.776 ***	11.316 ***	2.856 ns	14.571 ***
NPQ	128.041 ***	51.985 ***	12.940 ***	110.697 ***	8.295 **	14.543 ***	10.033 ***
ETR	47.651 ***	219.949 ***	170.919 ***	7.890 **	10.386 ***	3.288 ns	2.479 ns
Na^+	1457.442 ***	25041.592 ***	722.523 ***	1539.318 ***	222.805 ***	899.190 ***	248.201 ***
K^+	45.016 ***	2781.287 ***	3767.096 ***	354.281 ***	63.384 ***	87.351 ***	103.068 ***
Ca^{2+}	15.259 ***	1267.878 ***	642.867 ***	177.214 ***	18.349 ***	11.724 ***	25.468 ***
Mg^{2+}	26.050 ***	1342.470 ***	1653.453 ***	98.922 ***	73.894 ***	19.263 ***	46.564 ***
Na^+/K^+	1131.789 ***	11513.035 ***	575.535 ***	1315.140 ***	81.114 ***	439.588 ***	96.956 ***
Na^+/Ca^{2+}	1057.046 ***	8237.879 ***	270.484 ***	1123.866 ***	60.118 ***	243.307 ***	58.288 ***
Na^+/Mg^{2+}	506.302 ***	5173.002 ***	259.921 ***	606.199 ***	24.305 ***	193.233 ***	41.723 ***
Total saponin	105.517 ***	165.854 ***	13.199 ***	22.376 ***	4.095 *	10.881 ***	23.321 ***
Total flavonoids	92.551 ***	3.923 *	15.651 ***	37.547 ***	30.534 ***	27.957 ***	12.415 ***
Total phenols	3.828 ns	219.271 ***	46.791 ***	81.404 ***	40.150 ***	8.586 **	13.565 ***
CBD	23146.304 ***	217847.505 ***	159905.261 ***	6700.303 ***	968.271 ***	92494.107 ***	5011.859 ***

F-values are followed by *P*-values; ns *P* 0.05; **P* <0.05; ***P* <0.01; ****P* <0.001

Table S2. Growth parameters of hemp under salt stress and drought stress

	FW(g)		DW(g)		Height(cm)		SD(mm)		NB(pc)		RWC(%)	
	**NM**	**AM**	**NM**	**AM**	**NM**	**AM**	**NM**	**AM**	**NM**	**AM**	**NM**	**AM**
CK	34.8 ± 1.4b	37.0 ± 1.5a	11.1 ± 0.3a	11.8 ± 0.4a	87.7 ± 9.5b	88.4 ± 8.9a	6.68 ± 0.26b	7.52 ± 0.23a	15 ± 1b	18 ±1 a	77.5 ± 0.3cd	85.7 ± 2.0a
S1	22.7 ± 0.6g	34.3 ± 1.1bc	7.2 ± 0.2f	10.5 ± 0.4c	81.3 ± 3.8ab	84.4 ± 4.5ab	5.54 ± 0.26e	6.56 ± 0.13b	13 ± 1cde	13 ± 1cd	71.1 ± 1.0e	82.9 ± 2.5ab
S2	12.8 ± 0.4i	27.8 ± 0.4d	3.5 ± 0.2j	8.2 ± 0.1e	82.3 ± 4.7ab	74.2 ± 3.2bc	4.81 ± 0.05f	5.68 ± 0.28de	9 ± 1f	13 ± 1cd	65.5 ± 0.5fg	60.9 ± 0.8h
D1	19.8 ± 0.6h	33.2 ± 0.8c	6.7 ± 0.1g	10.3 ± 0.2c	83.8 ± 7.5ab	84.8 ± 7.0ab	5.82 ± 0.13cde	7.24 ± 0.34a	12 ± 1de	15 ± 1b	69.8 ± 0.7e	80.4 ± 5.5bc
D2	12.5 ± 0.9i	26.3 ± 0.3e	4.0 ± 0.2i	9.0 ± 0.1d	65.3 ± 2.6c	82.4 ± 6.4ab	4.98 ± 0.09f	6.02 ± 0.12cd	12 ± 1de	15 ± 1b	65.4 ± 1.3fg	75.3 ± 3.2d
S1D1	12.8 ± 0.2i	27.1 ± 0.4de	5.0 ± 0.0h	7.6 ± 0.3f	84.6 ± 6.45ab	82.5 ± 5.2ab	5.98 ± 0.1cd	6.63 ± 0.07b	11 ± 1e	14 ± 1bc	68.8 ± 1.1ef	75.0 ± 3.3d
S1D2	12.2 ± 0.8i	24.9 ± 0.5f	3.2 ± 0.2j	7.6 ± 0.4f	76.3 ± 10.3abc	72.0 ± 9.8bc	4.69 ± 0.11f	6.13 ± 0.22c	12 ± 1de	12 ± 1de	62.0 ± 0.6gh	68.2 ± 1.5ef

Values in the table represent mean ± S.E. Different letters indicate significant differences between hemp with or without AMF under each treatment.
